# EPA-lactone derivative, 5,6-diHETE lactone, improves pulmonary arterial hypertension in a monocrotaline-induced model

**DOI:** 10.3389/fphar.2025.1621030

**Published:** 2025-07-10

**Authors:** Karthik Dhananjayan, Offir Ertracht, Shaul Atar, Alejandro Livoff, Mona Shehadeh, Andrea Szuchman-Sapir

**Affiliations:** ^1^ The Laboratory of Vascular Signaling Research, MIGAL-Galilee Research Institute, Ltd., Kiryat Shmona, Israel; ^2^ Department of Nutrition Sciences, Tel-Hai College, Kiryat Shmona, Israel; ^3^ The Cardiovascular Research Laboratory, Research Institute, Galilee Medical Center, Nahariya, Israel; ^4^ The Cardiology Department, Galilee Medical Center, Nahariya, Israel; ^5^ The Azrieli Faculty of Medicine, Bar-Ilan University, Safed, Israel; ^6^ Department of Pathology, Galilee Medical Center, Nahariya, Israel; ^7^ Clinical Laboratories Division, Galilee Medical Center, Nahariya, Israel

**Keywords:** monocrotaline, pulmonary hypertension, 5,6-DiHETE, eicosapentaenoic acid, and inflammation respiratory pharmacology, vascular remodelling, and inflammation

## Abstract

**Background:**

Pulmonary arterial hypertension (PAH) is a progressive pulmonary arteriopathy characterized by vascular remodeling and subsequent increases in pulmonary vascular resistance, which further develops into right ventricular failure and death. Currently, PAH management targets pulmonary vasoconstriction, though there is an unmet medical need to develop therapeutics focusing on pulmonary vascular remodeling. Recently, we reported that 5,6-diHETE lactone (EPA-L, a stable metabolite of the EPA fatty acid) elicits vasodilation and blood-pressure-lowering effect in 5/6 nephrectomy hypertensive rats and vasodilation in human arterioles by an endothelial-dependent mechanism.

**Aim:**

We aimed to investigate the effect of EPA-L in a monocrotaline (MCT)-induced rat model of PAH.

**Methods:**

Sprague-Dawley Rats were divided into four groups; 3 received MCT (60 mg/kg, s. c.), and the control group was treated with saline. After 3 weeks, MCT rats were treated with saline, 0.3 or 3.0 mg/kg EPA-L, for five consecutive days. Finally, all animals were sacrificed upon functional, hematological, and histological evaluations.

**Results:**

EPA-L administration (i.v.) significantly reduced mean pulmonary arterial pressure (p < 0.05), echocardiographic pulmonary artery time-to-peak (p < 0.05), arterioles intimal-media thickness (p < 0.05) compared to the MCT group. Blood chemistry resulted in a significant reduction in hypoxic indices following the EPA-L administration, but it did not reduce the macrophage infiltration to the lungs and indicators of systemic inflammation, such as neutrophil count and % lymphocyte.

**Conclusion:**

In addition to the dilation properties, EPA-L attenuates MCT-induced pulmonary hypertension by improving hemodynamic parameters, and vascular modification. Therefore, EPA-L may act as a promising candidate for treating PAH.

## Graphical Abstract

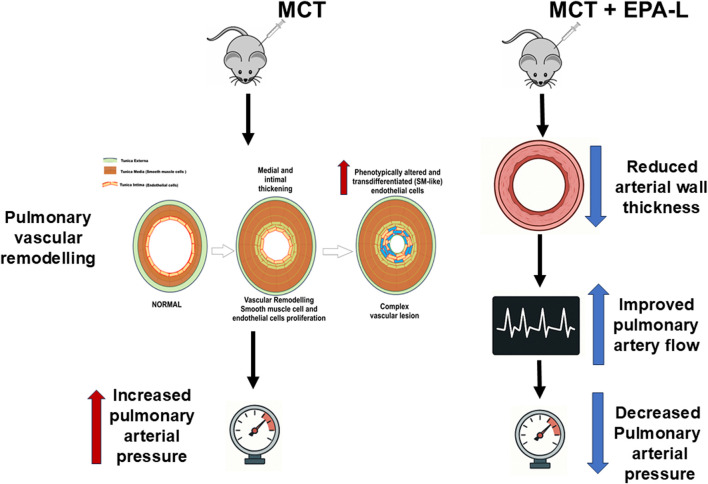

## 1 Introduction

Pulmonary arterial hypertension (PAH) is a life-threatening disease that causes increased pulmonary artery pressure, which eventually results in right heart failure and death if left untreated (Levine, 2021). The worldwide estimated prevalence of PAH ranges from 10 to 52 cases per million people, and incidence of 2–3 cases per million people per year (Valverde et al., 2018), with females aged 30–60 years more prone to PAH than males (Cheron et al., 2021). PAH is diagnosed through mean pulmonary artery pressure (mPAP) measurement >20 mmHg at rest (Levine, 2021; Cullivan et al., 2022). It is characterized by irreversible vascular remodeling, suggested to be initiated by vascular endothelial dysfunction, followed by smooth muscle cell proliferation, fibrosis, and the loss of vascular patency. Ultimately leading to vessel lumen narrowing, thickened and stiffer vessel walls, and increased resistance to blood flow. Over time, this heightened vascular resistance causes right ventricle hypertrophy and, eventually, failure.

Nowadays, PAH therapy targets pulmonary vasoconstriction by vasodilator administration, such as prostacyclin (e.g., Epoprostenol) (Ruan et al., 2010), soluble guanylate cyclase (sGC) stimulators (e.g., Riociguat) (Stasch and Evgenov, 2013), endothelin receptor antagonists (e.g., Bosentan) (Enevoldsen et al., 2020), calcium channel blockers (e.g., Diltiazem) (Medarov and Judson, 2015), and PDE5 inhibitors (e.g., Sildenafil citrate) (Galiè et al., 2005). Other treatments concentrate on blood clots prevention by anticoagulants, heart failure, and pulmonary edema prevention by digoxin and diuretics, respectively (Corris and Degano, 2014). Although available therapies for PAH have notably improved the survival of patients, a significant portion of patients do not achieve the expected efficacy. Thus, investigating the underlying mechanism of the development of PAH and new molecular targets that prevent pulmonary vascular remodeling is considered an unmet need.

Recently, we reported that 5,6-EEQ lactone (EPA-L), a stable lactone-metabolite derived from the CYP epoxygenase pathway of the EPA fatty acid, elicits a blood-pressure-lowering effect and microvessels dilation in 5/6 nephrectomy hypertensive rats (Barsheshet et al., 2021) and human arterioles [12]. It was shown that its cellular mechanism of action depends on the endothelial PLC-IP3-signaling pathway that activates potassium efflux, resulting in endothelial hyperpolarization. The EPA-L vascular dilation was shown to be independent of nitric oxide in hypertensive human microvessels (Asulin et al., 2024). Thus, to explore whether the EPA-L may also improve vascular dysfunction in pulmonary hypertension, we investigated the hemodynamic and histological effects in a monocrotaline-induced PAH rat model.

## 2 Materials and methods

### 2.1 Chemicals

Monocrotaline, 3,3′-Diaminobenzidine, Picric acid, Direct-red 80, acetic acid glacial, and 30% hydrogen peroxide (Sigma-Aldrich, Ltd, Rehovot, Israel), EPA-lactone ((±)5,6-DiHETE Lactone, Toronto Research Chemicals (TRC), Holland-Moran, Israel), CD68 (Santa Cruz Biotechnology, Inc. USA), Goat anti-rabbit HRP conjugate (Bio-Rad, USA). All the solvents used are of HPLC grade.

### 2.2 Animals

All animal experiments were conducted according to the institutional animal ethical committee guidelines (ethical number: BIU - M.D. - IL - 2302 - 107 - 4), which conform to the Guide for the Care and Use of Laboratory Animals published by the U.S. National Research Council (Eighth edition 2011). The number of animals needed for the study was calculated using 12.0 Sigma-Plot (Palo Alto, CA, USA). Male Sprague-Dawley (217 ± 52 g) rats (Envigo Ltd, Jerusalem, Israel) were maintained at a constant temperature and relative humidity under a regular light/dark schedule (12:12), fed with normal rodent diet and tap water *ad libitum*.

### 2.3 Study design

All rats were purchased from an external authorized animal supplier (Harlan LTD, Israel). After several days of acclimation, each rat was randomly chosen to undergo its designated treatment profile. The pre-planned protocol dictated each rat’s experimental profile, including the exposure of a rat to either saline (control) or MCT and the treatment: saline, or one of two doses of EPA-Lactone (0.3 or 3 mg/kg). Rats were divided randomly into four experimental groups (n = 4-7 in each group): A. Control; B. Monocrotaline (MCT) injected; C. MCT injected +0.3 mg/kg EPA-L treatment; D. MCT injected +3 mg/kg EPA-L treatment.

**FIGURE 1:**
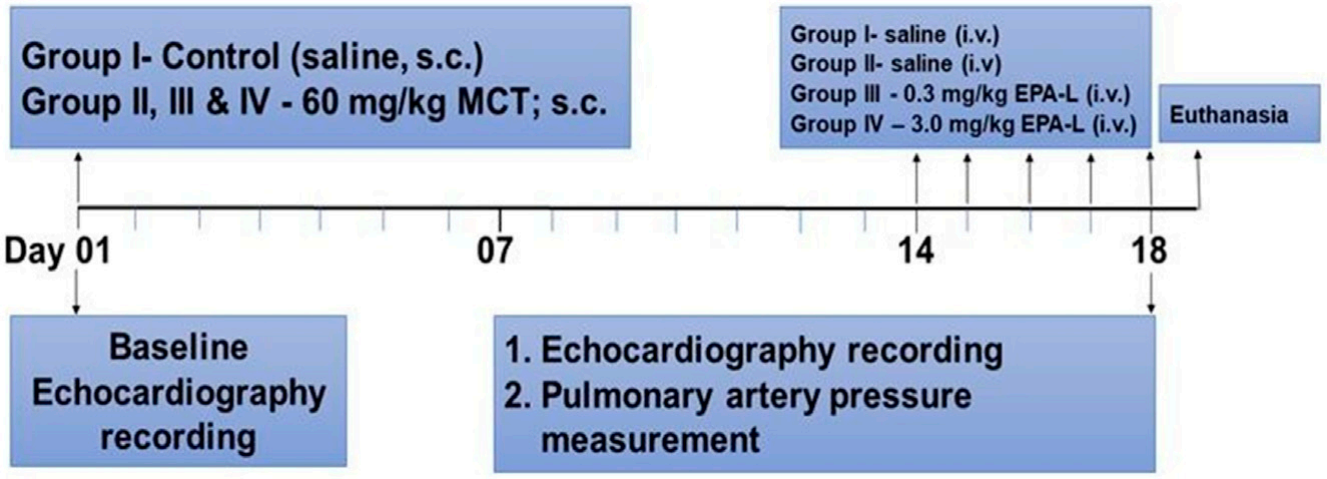
Schematic representation of study design.

At baseline, body weight (BW) was recorded, and general phenotypic evaluation. Then each rat underwent echocardiography under light sedation with Ketamine (29 mg/kg) and Xylazine (4.3 mg/kg). Rats of group A were injected with 0.5 mL saline S.C., and the rest of the animals were administered with 60 mg/kg MCT dissolved in HCl (1N), diluted with distilled water, and administered after adjusting the pH to 7.4. Two weeks later, after weight and echocardiography evaluation, each day from 14 to 19 (five consecutive days), rats of groups A and B were injected with 0.5 mL saline I.V. Groups C and D were treated with 0.3 mg/kg/day and 3 mg/kg/day EPA-L dissolved in 0.5 mL saline, respectively. On the last day (#19), each rat underwent final BW, echocardiography assessment, and direct measurement of mPAP. Blood was then withdrawn from the heart and sent to complete blood count (CBC) and biochemical analyses. At the end of the experiment, animals were sacrificed by anesthesia overdose, and KCl (1M) was used to stop the heart at diastole. The lungs were harvested and preserved in 4% paraformaldehyde for later analyses (Figure 1).

### 2.4 Echocardiography

For the echocardiographic measurements, rats were lightly sedated by an intra-muscular (I.M.) injection of 29 mg/kg ketamine and 4.3 mg/kg xylazine. After sedation, the rats were placed in a left decubitus position and scanned via a commercially available echo-scanner (Vivid *I*, GE, Israel), using a 10S phased array pediatric transducer with a cardiac application. The transmission frequency was 12 MHz, the depth was 2.5 cm, and the frame rate was at least 315 frames/sec. Acquisition and analyses of continuous wave (C.W.) Doppler scans were used to portray characteristic pulmonary artery flow velocity profiles, from which peak flow velocity (Vmax) and time to peak (TTP) in the pulmonary artery were estimated to characterize PAH. All echocardiographic acquisitions were performed by the same operator using a standardized protocol. The operator followed a strict, standardized imaging procedure for every animal (consistent animal positioning, identical ultrasound settings, and landmarks for measurement) (Koskenvuo et al., 2010).

### 2.5 Right heart catheterization and measurement of mean pulmonary arterial pressure (mPAP)

For direct mPAP measurement, each rat was deeply anesthetized (Ketamine 87 mg/kg + Xylazine 13 mg/kg) and was given analgesia (Buprenorphine 0.05 mg/kg). The rat was intubated and ventilated at 80–90 breaths per minute and a tidal volume of 1–2 mL/100 gr BW. The rat’s chest was opened on the right side at the third or fourth intercostal space. A 21G needle attached to a DTX-1 pressure transducer (Cwe, Inc Ardmore, PA, U.S.A.) attached to a TA-100 amplifier (Cwe, Inc Ardmore, PA, U.S.A.) was used to puncture the pulmonary artery and to record directly the pulmonary artery pressure for 2–5 min. The pressure transducer underwent two-point calibration before right heart catheterization with a mercury manometer; further upon insertion into the animal, the transducer was calibrated to 0 mmHg at the height of the right atrium before measurements to ensure accuracy.

### 2.6 Blood analyses

CBC data included, among others, parameters related to hypoxia (i.e., red blood cell size, haemoglobin concentration, mean capsular haemoglobin concentration, mean capsular volume, and total protein in the whole blood). In the CBC, we have also obtained inflammation levels (i.e., number and distribution of white blood cells and their different sub-classifications, mainly neutrophils and lymphocytes). CBC was conducted by Sysmex XN-1000™ Hematology Analyzer (Sysmex America, Inc. Lincolnshire, IL, USA). Serum was obtained by whole blood centrifugation at 4°C, 2500Xg, for 10 min and analyzed using the Alinity systems (Abbott Core Laboratory Systems, Lake Forest, IL, USA).

### 2.7 Histology

The lung tissues underwent standard blocking and paraffin embedding. Then, the blocks were sectioned to 5 µm using (Microtome, Thermo Scientific, Cheshire, United Kingdom), and mounted on histological slides. Slides were stained either with hematoxylin-eosin (H&E) or Picro-Sirius red (for collagen staining).

#### 2.7.1 Hematoxylin and eosin (H&E) staining

Briefly, upon de-paraffinization and re-hydration, slides were stained with hematoxylin Gill III for 8 min and washed with tap water. Then, the tissue slides were dipped in 1.5M HCl (4 s), tap water (4 min), 95% ethanol (2 min), and alcoholic eosin Y (40 s). Finally, the slides underwent dehydration (100% alcohol followed by Xylene) and were mounted with DPX Mount and covered with coverslips.

#### 2.7.2 Picro sirius red staining

The de-paraffinized and re-hydrated slides were treated with Weigert’s iron Hematoxylin solution for 8 min and washed with running tap water for 10 min. Next, the slides were stained with Picro-Sirius red solution for 1h, followed by 0.5% acidified water twice. Finally, it was dehydrated with ethanol (100%) and xylene, mounted by DPX mount, and covered with coverslips.

#### 2.7.3 Histological analyses

Stained slides were visualized at a magnification of×10, ×20, or x40 and captured in a bright field under an Olympus IX73 microscope. Wall thickness and wall-lumen ratio were determined from the diameter (internal and external) of the arterioles (50–150 µm) using Equations 1, 2, respectively:

$$Wall\;thickness\;\left(WTh\right)=\frac{ED\;\left(µm\right)-ID\left(µm\right)}{2}$$ (1)

$$Wall-lumen\;ratio\;\left(WLR\right)=\frac{WTh\;}{Internal\;diameter\;}$$ (2)

Where, ED = External diameter; ID = Internal diameter.

The Sirius-red stained collagen area was measured in lung tissue under the microscope (Nikon Eclipse Ci-L, Nikon Corporation, Tokyo, Japan) using the NIS Elements software (NIS Elements 4.0, Nikon Corporation, Tokyo, Japan). For each animal, 5 fields were photographed (×100 magnification). In each picture, the collagen (red) area and the total tissue area were measured, and the collagen volume fraction (CVF) was calculated from the % of collagen area divided by total tissue area.

### 2.8 Immunohistochemistry

Deparaffinized tissue slides were immersed in citric acid buffer (10mM, pH = 6) for antigen retrieval at 90°C for 10 min. The slides were washed with PBS and treated with 3% hydrogen peroxide (H_2_O_2_). Followed by subsequent washing with PBS, the non-specific areas were blocked by incubation with 2% bovine serum albumin (BSA) for 1h at R.T. Then the tissue slides were treated with 150 µL of primary antibody (CD68, Santa Cruz (KP1) Dallas, Tx, USA; 1:50) and incubated overnight at 4°C. The tissue slides were washed with 10% PBST (phosphate buffered saline + tween 20) and incubated with a secondary antibody (Donkey Anti-mouse HRP conjugate, Bethyl Laboratories, Inc, Tx, USA; 1:50) for 20 min at room temperature. For SMAD3 detection, tissue slides were incubated with 150 µL of primary antibody (SMAD3, Abcam Limited, Cambridge, United Kingdom); 1:200) and incubated overnight at 4°C. The tissue slides were washed with 10% PBST (phosphate buffered saline with tween 20) and incubated with secondary antibody (Donkey Anti-mouse HRP conjugate, Bethyl Laboratories, Inc, Tx, USA; 1:50) for 20 min at R.T. All slides were then re-washed with PBST and incubated with 1% DAB (3,3′ diaminobenzidine) chromogen in DAB buffer solution for 30 min at R.T. Finally, the slides were washed in running water and counterstained with Hematoxylin for 15 s, then with PBS, and dehydrated using a series of solvents (70% ethanol, 96% ethanol, 100% ethanol, and xylene). Slides were mounted with mounting medium and covered with coverslips. Images were taken using an Olympus IX73 microscope under a bright field (x10 or x20), and the localization of macrophages was quantified by counting the number of positive cells as a percentage of the total number of cells counted, using the IHC tools plugin installed in ImageJ software Version 1.54D (NIH, USA).

### 2.9 Lipidomic analyses using ultra-performance liquid chromatography coupled mass spectrometry/mass spectrometry (LC-MS/MS)

The plasma levels of diverse lipid metabolites were determined by MetaToul- Lipidomics (INSERM, France) using LC-MS/MS. Two-60 μL of cold methanol and 40 µL of internal standard (Deuterium labeled) were added to 100 µL of plasma in a 1.5 mL Eppendorf tube for deproteinization. Post-centrifugation at 5000 *g* for 15 min at 4°C, supernatants were transferred to 96-well plates and diluted in H_2_O to 2 mL. Samples were then submitted to solid phase extraction (SPE) using an OASIS HLB 96-well plate (30 mg/well, Waters) pretreated with MeOH (1 mL) and equilibrated with 10% MeOH (1 mL). After sample application, the extraction plate was washed with 10% MeOH (1 mL). Finally, samples were dried under aspiration, and lipids mediators were eluted with 1 mL of MeOH. Prior to LC-MS/MS analysis, samples were evaporated under nitrogen gas and reconstituted in 10 µL of MeOH.

LC-MS/MS analyses of eicosanoids were performed as described (Le Faouder P et al., 2013). All lipidomic quality control criteria were fully satisfied according to The QQ report from the lipidomic facility core at MetaToul-Lipidomique (I2MC, Inserm, Toulouse, France), MetaboHUB-ANR-11-INBS-0010. The LC-MS/MS instrument was tuned and calibrated, with no indication of carryover or contamination–enzymatic oxylipins were not detected in blank (negative control) samples (signal-to-noise <3). All deuterated internal standards (LxA4-d5, LTB4-d4, and 5-HETE-d8) were consistently detected in the calibration and biological samples, confirming stable instrument performance throughout the analyses. The method exhibited retention time repeatability (day-to-day variation ΔRT <0.2 min), and the external calibration curves for all analytes showed linearity ofR^2^ ≥ 0.99). The lipidomic analysis was performed using a validated LC-MS/MS protocol (Le Faouder et al., 2013).

Lipid mediators (reference standards) were separated on a ZorBAX SB-C18 column (2.1 mm, 100 mm, 1.8 µm) (Agilent Technologies) using Agilent 1290 Infinity HPLC system (Technologies) coupled to an ESI-triple quadruple G6460 mass spectrometer (Agilent Technologies). Data were acquired in Multiple Reaction Monitoring (MRM) mode with optimized conditions (ion optics and collision energy). Peak detection, integration and quantitative analysis were done using Mass Hunter Quantitative analysis software (Agilent Technologies) based on calibration lines built with commercially available eicosanoids standards (Cayman Chemicals). Accordingly, the quantity of each metabolite was determined (pg/µL plasma) for each treatment, and the relative change at the end of the experiment was calculated from the average concentrations at baseline using the (final-baseline)/baseline formula.

### 2.10 Statistical analysis

Data were analysed using one-way ANOVA, where the group was the independent parameter. When ANOVA was significant, the *post-hoc* Dunnett’s test was used. For repeated measures, two-way ANOVA was used, where time and groups were the independent parameters. For parameters measured with low sample size (n < 5), the Kruskal–Wallis test for non-parametric statistical test was used. p values of <0.05, were considered significant.

## 3 Results

**FIGURE 2:**
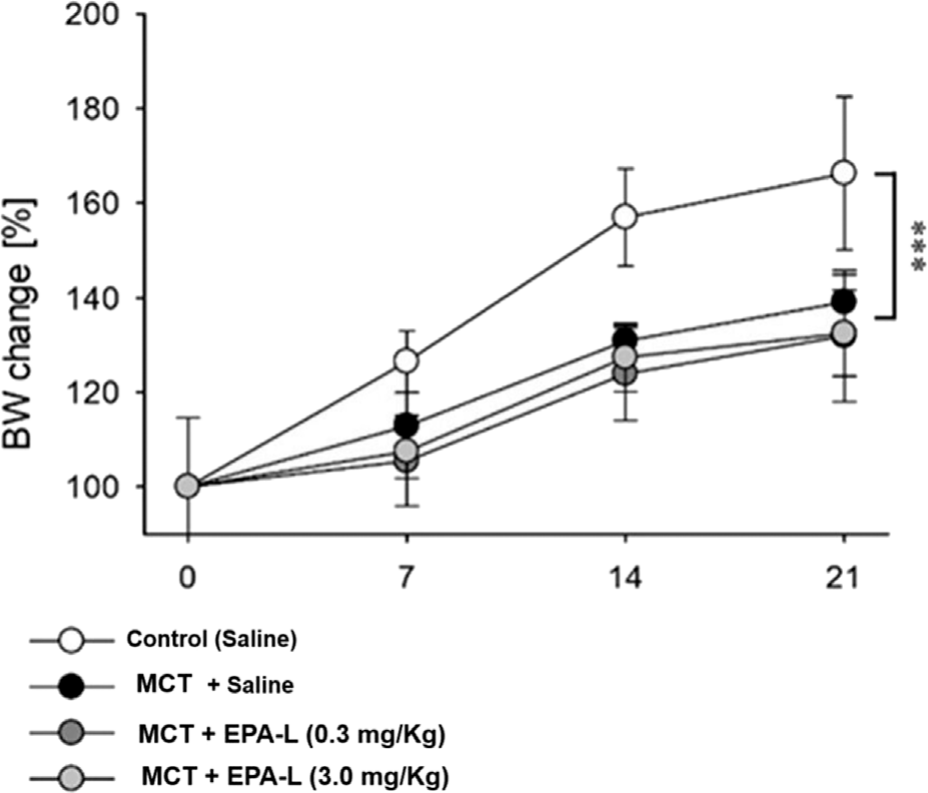
Percent change of body weight during the experiment. Body weight (BW) change was calculated as the percent of basal BW for each rat and averaged for each time point and group. In 2 Way ANOVA, a significant difference (P < 0.001) was found between the saline-treated group vs. the other groups (monocrotaline-treated groups).

At baseline, BW were 172.5 ± 5.3, 229.8 ± 4.9, 181.0 ± 4.8, and 174.5 ± 4.4 g for groups A, B, C and D, respectively. The BW increase was attenuated in all MCT-treated rats (groups B-D), while the saline-treated group (group A) kept normal BW gaining (Figure 2, p < 0.001) along the experimental time. The water content of the lungs (%) was determined and calculated as 79.37% ± 1.83% for normal control vs. MCT rats (81.11% ± 0.38%). In MCT + EPA-L treated rats, the water content of the lungs (%) was 80.63% ± 0.72% and 82.97% ± 1.72% for 0.3 and 3.0 mg/kg EPA-L, respectively (p > 0.05).

### 3.1 EPA-L improves haemodynamic measurements

**FIGURE 3:**
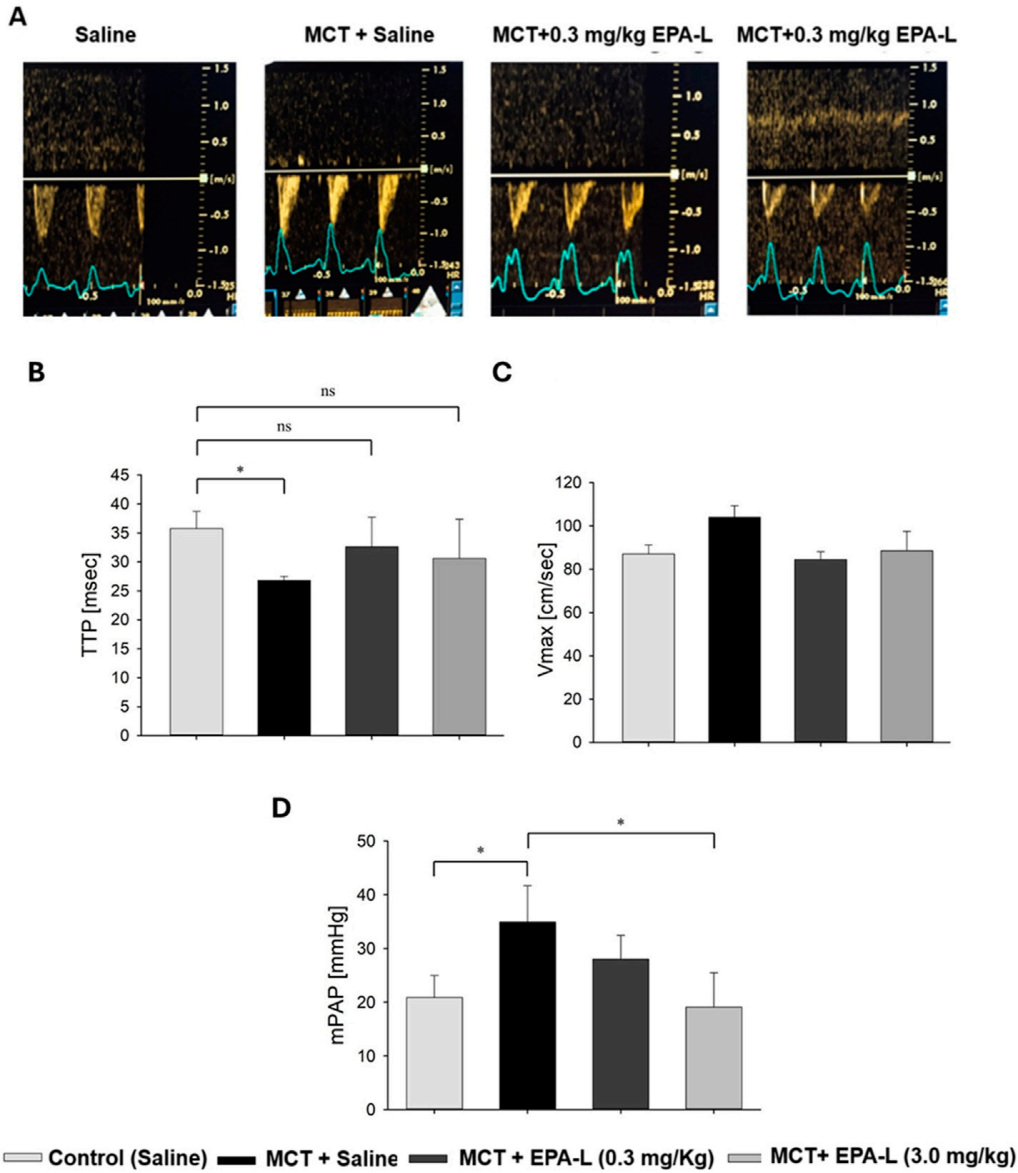
Echocardiography results. **(A)** Representative pulsed wave Doppler lines of pulmonary arteries in rats treated with (left to right) saline (control), MCT + saline, and MCT + EPA-L in two concentrations (0.3 and 3 mg/kg). **(B)** The TTP was averaged from six beats on day 1 and the final day of the experiment, n = 3-7 rats per treatment. **(C)** Vmax was averaged from six beats on day 1 and the final day of the experiment, n = 3-7 rats per treatment. **(D)** Mean pulmonary arterial pressure (mPAP). n = 3-5, non-parametric One-way ANOVA with Kruskal–Wallis multiple comparisons test *p < 0.05.

The MCT challenge significantly reduced the TTP in the saline-treated group (35.3 ± 3.9 msec) to 26.8 ± 0.7 msec (p < 0.05), while the addition of 0.3 mg/kg and 3 mg/kg EPA-L post-MCT moderated TTP reduction (32.6 ± 5.1 msec, and 30.6 ± 6.8 msec, respectively) (p < 0.05) (Figure 3B), similar to the saline-treated group. Concomitantly, although non-significantly, basal Vmax (87 ± 8 cm/s) that was preserved in the saline-treated rats (87 ± 4 cm/s), was increased to 104 ± 5 cm/s in the MCT alone group, while reduced back to 85 ± 4 cm/s in the 0.3 mg/kg EPA-L treated group and to 89 ± 9 cm/s in the 3 mg/kg EPA-L treated group (Figure 3C).

### 3.2 EPA-L reduced mean pulmonary artery pressure (mPAP)

The analysis of mPAP showed significant differences among the groups. In the MCT-treated rats, the mPAP (33.9 ± 3.0 mmHg) significantly increased compared to the saline-treated rats (20.9 ± 2.2 mmHg, p < 0.05). Treatment with 0.3 mg/kg EPA-L decreased the mPAP by 19.7% (28.0 ± 2.5 mmHg, p > 0.05), and treatment with 3 mg/kg EPA-L reduced the mPAP (19.07 ± 3.21mmHg, p < 0.05) abolishing the MCT-treated mPaP increase (Figure 3D).

### 3.3 Effect of EPA-L on systemic hypoxia

**FIGURE 4:**
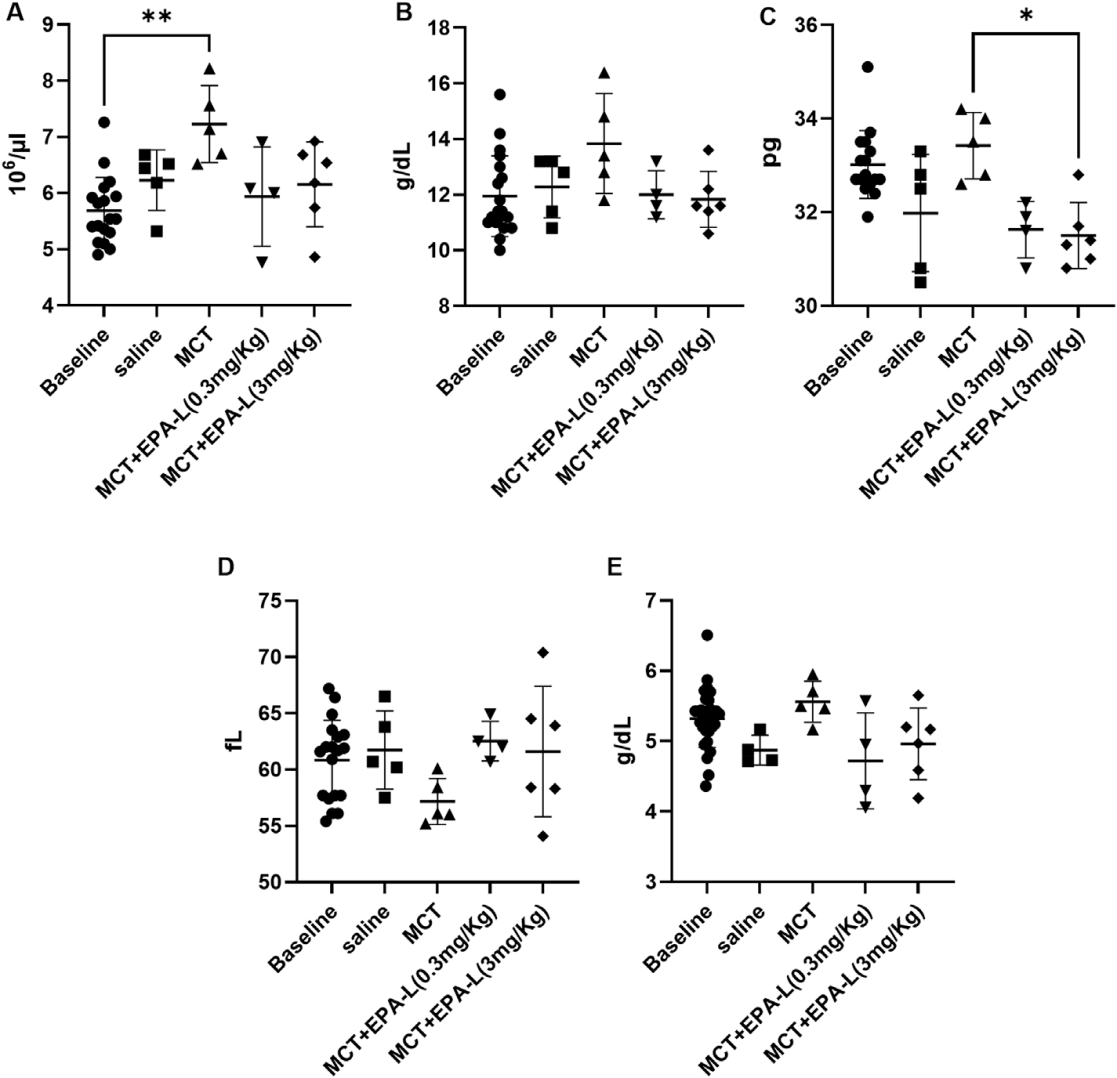
Haematological parameters. **(A)**. Red blood cell count **(B)**. Haemoglobin concentrations **(C)**. Total protein **(D)**. Mean corpuscular haemoglobin concentrations **(E)**. Mean corpuscular volume. n = 4; non-parametric One-way ANOVA with Kruskal–Wallis multiple comparisons test, *p < 0.05; **p < 0.01.

Haematological parameters RBC, HGB, MCHC, and MCV for the saline-treated rats at the basal state (5.68 ± 0.60 10^3^×µl^-1^, 11.95 ± 1.45 g/dL, 20.95 ± 1.60 g/dL, and 60.85 ± 3.54 fL, respectively) were comparable to their final state (6.23 ± 0.54 10^3^×µl^-1^, 12.28 ± 1.11 g/dL, 19.74 ± 1.18 g/dL, and 61.74 ± 3.48 fL, respectively (Figures 4A–D). The MCT-treated rats had increased levels of RBC (7.23 ± 0.69 10^3^×µl^-1^, P < 0.01) compared to baseline (BL) (Figure 4A). HGB, MCHC, and TP (13.84 ± 1.80 g/dL, 33.4 ± 0.7 g/dL, 5.6 ± 0.3 g/dL, respectively) were comparable to baseline and saline, and MCV was moderately reduced (57.16 ± 2.03 fL) compared to all groups (Figures 4B–E).

After treatments with 0.3 and 3.0 mg/kg EPA-L, the RBC levels (5.94 ± 0.88 103×µl^-1^, 6.15 ± 0.76 10^3^×µl^-1^, respectively), HGB levels (12.00 ± 0.86 g/dL, 11.83 ± 1.01 g/dL, respectively), MCV levels (62.53 ± 1.56 fL, 61.60 ± 5.80 fL, respectively), and TP (4.72 ± 0.68 g/dL, 4.96 ± 0.51 g/dL, respectively) were comparable to all groups (Figures 4A,B, and 4D-4E). While, MCHC levels were significantly reduced after EPA-L treatment of the higher dose (31.5 ± 0.71 g/dL, p < 0.05) (Figure 4C).

### 3.4 EPA-L reduced the wall-thickness and wall-lumen ratio

**FIGURE 5:**
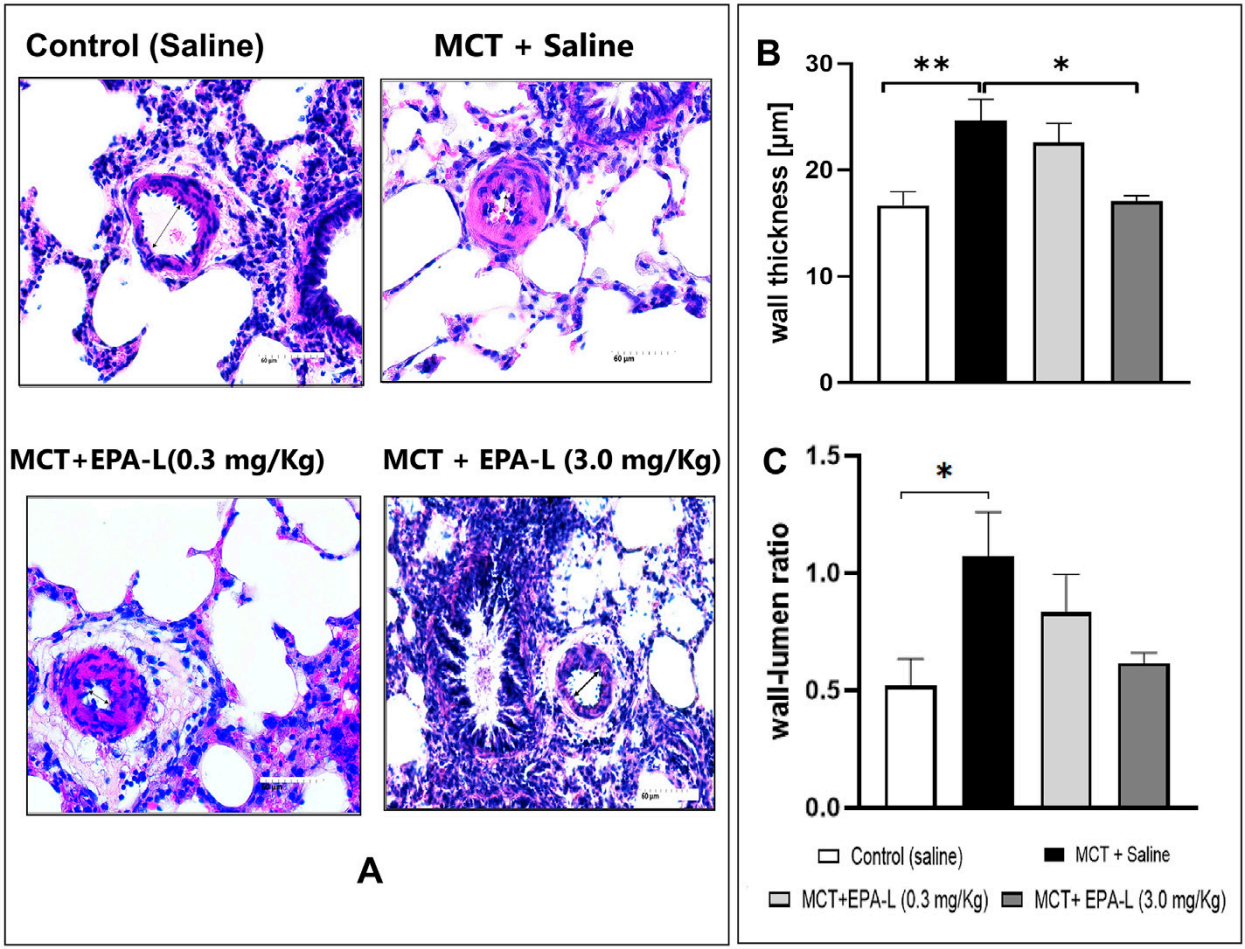
Measurement of intimal media thickness and wall-lumen ratio. **(A)** H&E staining of lung tissues visualized under 40X. The arterioles 50–150 µm in diameter were examined **(B)** Wall thickness, 3.0 mg/kg EPA-L significantly decreased the SMC proliferation vs. MCT, **(C)** wall-lumen ratio. n = 3-5; non-parametric One-way ANOVA with Kruskal–Wallis multiple comparisons test, *p < 0.05, **p < 0.01.

The wall thickness analysis showed significant differences among the groups. The wall thickness of saline-treated MCT rats significantly (p < 0.01) increased (24.69 ± 6.13 µm) versus saline-treated normal control rats (16.63 ± 3.79 µm). In MCT-rats treated with 0.3 mg/kg EPA-L, there were no significant differences in the wall thickness (22.59 ± 5.36 µm) compared to saline-treated MCT rats, whereas MCT-rats treated with 3.0 mg/kg EPA-L showed significant decreases (17.04 ± 1.37 µm, p < 0.05) in wall thickness versus saline-treated MCT rats (Figures 5A,B).

The analysis of the wall-to-lumen ratio showed differences among the groups. The wall-lumen ratio increased significantly by 2.05-fold in saline-treated MCT rats (1.07 ± 0.59 µm, p < 0.05) compared to saline-treated normal control rats (0.52 ± 0.32 µm). The wall-lumen ratio decreased by 0.77 and 0.57-fold in MCT-rats treated with 0.3 mg/kg EPA-L (0.83 ± 0.50 µm) and MCT-rats treated with 3 mg/kg EPA-L (0.61 ± 0.09 µm) versus saline-treated MCT rats (Figure 5C), although not significantly.

### 3.5 Effect of EPA-L on fibrosis: collagen deposition and SMAD3 in lungs

**FIGURE 6:**
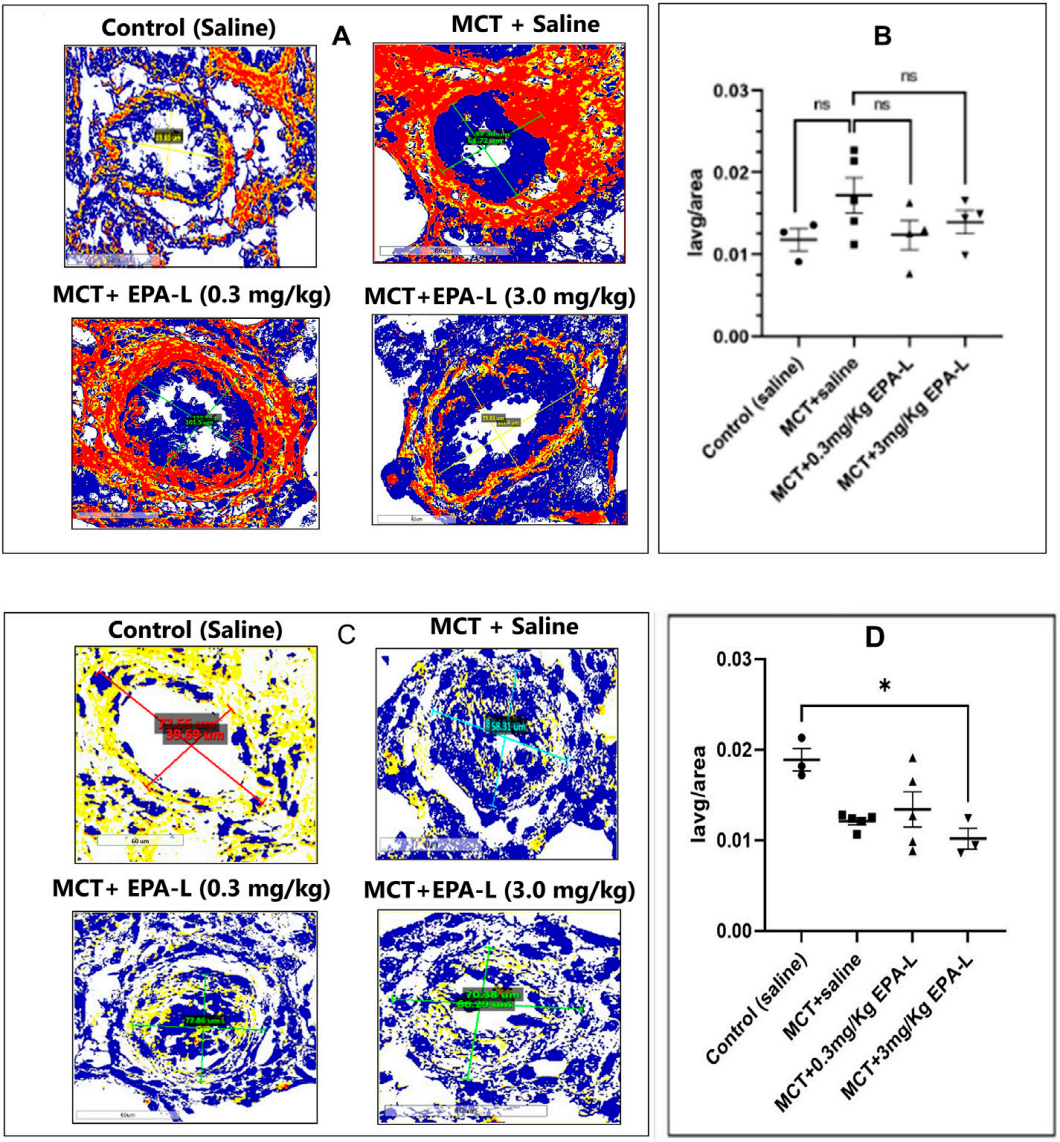
Tissue fibrosis: collagen deposition and SMAD3 in lungs. **(A)** Collagen deposition around the arterioles. The lung tissues were stained with picrosirius red and viewed under ×40 magnification, scale = 60 µm. **(B)** A plot of CVF on the y-axis against each group on the x-axis **(C)** SMAD3 expression around the arterioles. Immunohistochemistry of tissue stained with SMAD3 antibody and viewed under ×40 magnification, scale = 60 µm. **(D)** A plot of Iavg/tissue area on the y-axis against each group on the x-axis. Both CVF and SMAD3 images were viewed using Aperio Image Scope Software, the area around the arteriole was annotated, and the Iavg/area was calculated. n = 4; non-parametric One-way ANOVA with Kruskal–Wallis multiple comparisons test, *p < 0.05, ns = not significant. (Note: Yellow–Weak positive; Orange–Positive; Maroon- Strong positive; Blue–Negative).

The deposition of collagen measured as collagen volume fraction (CVF) increased around the arterioles insignificantly in the MCT-treated rats (0.017 ± 0.002) compared to saline-treated control rats (0.011 ± 0.002). Yet, treatment of MCT rats with 0.3 and 3.0 mg/kg EPA-L decreased CVF moderately around the arterioles by 0.012 ± 0.001 and 0.013 ± 0.001, respectively (Figures 6A,B) although statistically insignificantly.

The SMAD3 expression decreased in all MCT-treated rats (0.012 ± 0.00, p < 0.05), yet only in the 3.0 mg/kg EPA-L it reached statistical significance (P < 0.05) compared to saline-treated rats (0.018 ± 0.002) (Figures 6C,D).

### 3.6 Effect of EPA-L on indicators/markers of inflammation

**FIGURE 7:**
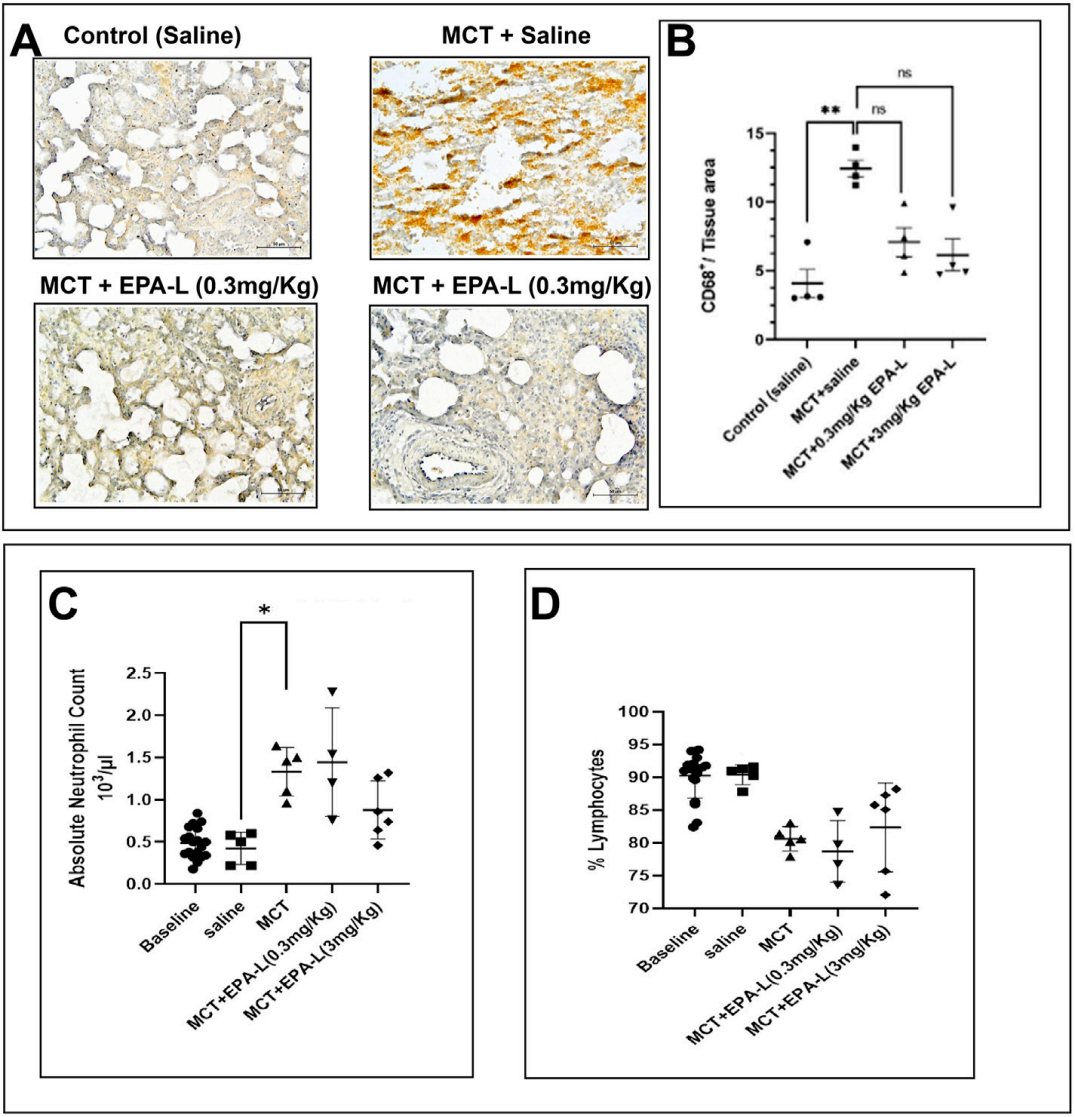
Macrophages invasion and circulating leukocytes. **(A)** CD68^+^ marker–The lung tissue sections were stained with CD68 antibody and counterstained with Hematoxylin. The images were captured under ×40 magnification, scale = 60 µm. The images were analysed using ImageJ 1.54d. The black arrow indicates the CD68^+^ cells **(B).** A plot of CD68^+^ per tissue area on the y-axis against corresponding groups on the x-axis. n = 4; non-parametric, **p < 0.01, ns = not significant. **(C)** Absolute neutrophil blood count **(D)** Lymphocyte percentage. n = 4; The absolute neutrophil count and % lymphocytes were analysed using One-way ANOVA with Kruskal–Wallis multiple comparisons test. **p < 0.01, ns = not significant.

Herein, the effect of EPA-L in MCT rats on markers/indicators of inflammation, such as CD68 (a surface marker of MQ) in lung tissue, plasma levels of neutrophils, and lymphocytes, was measured. In saline-treated MCT rats, CD68^+^ MQs increased significantly (12.45 ± 0.58, p = 0.0084) compared to saline-treated control rats (4.08 ± 1.00). The treatment of 0.3 and 3.0 mg/kg EPA-L decreased CD68^+^ MQs insignificantly by 0.56–folds (7.08 ± 1.07, p = 0.5258) and 0.49-folds (6.14 ± 1.15, p = 0.1554) respectively, compared to saline-treated MCT rats (Figures 7A,B).

Out of immune cells, the absolute neutrophil count (ANC) increased significantly in MCT rats (1.33 ± 0.12 × 10^3^ μL, p < 0.05) compared to saline-treated rats (0.42 ± 0.08 × 10^3^ μL). Whereas MCT rats treated with 0.3 and 3.0 mg/kg EPA-L showed 1.44 ± 0.32 × 10^3^ μL and 0.88 ± 0.13 × 10^3^ μL ANC, respectively, (p > 0.05 compared to all groups). The levels of lymphocytes were comparable among the groups. In the MCT rats, 80.62% ± 0.83% in the control rats, 90.40% ± 0.68%; in the 0.3 and 3.0 mg/kg EPA-L treated rats, 78.73 % ± 2.36% and 82.37% ± 2.75%, respectively, (Figures 7C,D).

### 3.7 Effect of EPA-L on ω-6 PUFA and ω-3 PUFA derived metabolites

**FIGURE 8:**
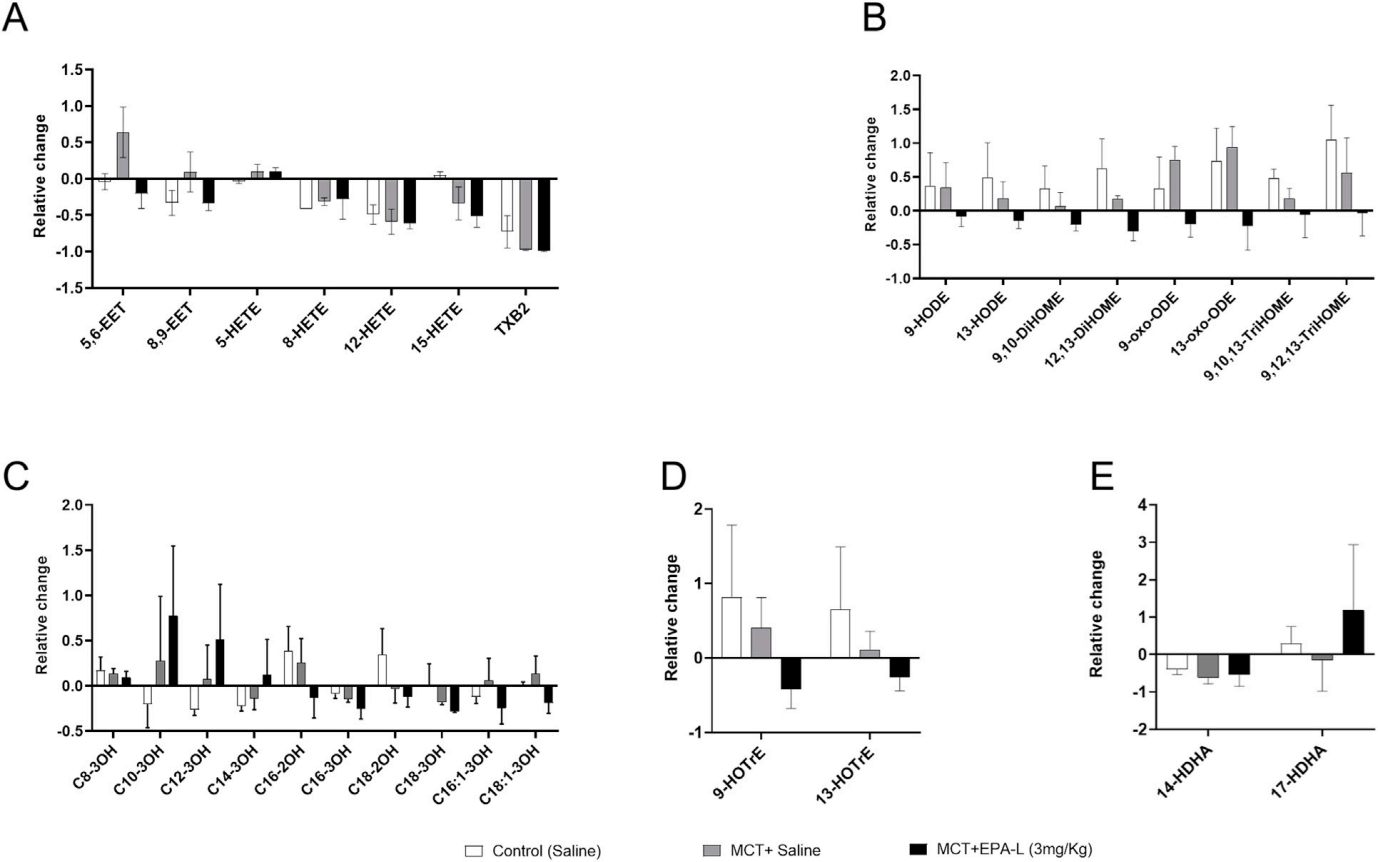
Targeted lipidomics–Relative change in the levels of ω-6 and ω-3 PUFA-derived metabolites. **(A)** Arachidonic acid (AA)-derived metabolites **(B)** Linolenic acid (LA)-derived metabolites **(C)** Hydroxy fatty acid metabolites **(D)** Docosahexaenoic acid (DHA) -derived metabolites **(D)** α-linoleic acid (ALA)-derived metabolites. Relative levels were calculated from each treatment’s concentrations at (final–baseline)/baseline. n = 9 for baseline, n = 3 for treatments; were analysed using One-way ANOVA with Kruskal–Wallis multiple comparisons test.

To identify valuable functional lipids that were characteristically altered by the EPA-L in the PAH-induced rats, we performed comprehensive lipidomic analysis using liquid chromatography-tandem mass spectrometry (LC-MS/MS). We determined the free PUFA metabolites in the blood samples of the rats at baseline and after the treatment with saline, MCT, or MCT following treatment with 3 mg/kg EPA-L. Among the 56 analysed metabolites (Listed in Supplementary Figure S1), the following metabolites’ relative levels were changed between the treatments: in the ω-6 PUFA class, the relative levels of the arachidonic acid (AA) metabolite- 5,6-EET were increased in the MCT-rats (0.64 ± 0.6) compared to the relative levels in the saline-treated rats (−0.04 ± 0.2). Treatment with 3 mg/kg EPA-L for 5 days reduced the 5,6-EET (−0.20 ± 0.3) and attenuated the 8,9-EET relative levels. Within the linoleic acid (LA) derived metabolites, EPA-L treatment reduced all the detected metabolites in comparison to the MCT-rats, notably the reduction in the relative levels of 13-oxo-ODE (0.93 ± 0.5 vs. −0.22 ± 0.6) and 9,12,13-TriHOME (0.56 ± 0.8 vs. −0.03 ± 0.5). Analysis of the hydroxy FA metabolites showed that EPA-L treatment moderately reduced the long-chain metabolites C:16-C:18 compared to the MCT rats, though it did not reach a significant value. Among the ω-3 PUFA class, the ALA-metabolites 9-HOTrE and 13-HOTrE were moderately reduced after the EPA-L treatment, while the DHA metabolite 17-HDHA relative levels were moderately elevated in comparison to the MCT-treated rats (1.174 ± 2.4 vs. −0.15 ± 1.1). No EPA metabolites were detected in the blood samples (Figures 8A–E).

## 4 Discussion

The effects of EPA-L on monocrotaline-induced pulmonary arterial hypertension in Sprague-Dawley rats were evaluated. The main finding points to a significant improvement in the lungs and heart hemodynamic measurements (mPAP, TTP, and Vmax) and blood hypoxic parameters in the MCT-induced PAH rats following treatment with the EPA-L. In addition, the administration of EPA-L significantly reduced microvascular wall thickness in the MCT-induced PAH lungs. The increase in inflammation in the PAH-induced rats, however, was not affected by the EPA-L, although an attenuation in macrophage invasion to the lungs was observed.

The mechanism by which MCT causes rat PAH involves liver metabolites of MCT damaging the pulmonary arterial endothelium. Endothelial dysfunction causes abnormal proliferation of pulmonary arterial smooth cells and excessive apoptosis of pulmonary arterial endothelial cells (Gomez-Arroyo et al., 2012; Hill et al., 2017). The remodeled pulmonary vasculature will then cause an elevation in pulmonary arterial pressure and right ventricular systolic pressure. In the present study, EPA-L was used to intervene in the late stage of the disease process to explore the therapeutic effect of EPA-L on PAH, and two doses of EPA-L were selected based on prior efficacy data, and to minimize animal usage.

Clinically, TTP and mPAP are primarily used to diagnose the incidence of PAH (Dammassa et al., 2022). The TTP measures the interval between the onset and peak of pulmonary artery blood flow (Granstam et al., 2013). Vmax, as an additional parameter, shows the increase of blood velocity within the pulmonary artery due to the increased stiffness of the arteries, and it correspondingly changes as the TTP. Thus, TTP, Vmax, and mPAP are used to study the changes in right ventricular performance and pulmonary vascular resistance (Dammassa et al., 2022). Clinical TTP is normally >130 m, and a TTP of <100 m indicates a high probability of PAH (Granstam et al., 2013). In our study, the TTP Vmax and mPAP of rats treated with a high dose of EPA-L (MCT +3 mg/kg EPA-L) were within the normal levels, which indicated EPA-L preserved the blood pressure against MCT-induced vascular resistance. These outcomes also align with our previous findings, where EPA-L elicited vasodilation and blood-pressure-lowering effects in 5/6 nephrectomy hypertensive rats (Barsheshet et al., 2021). The EPA-L effects can be further compared to reported outcomes of standard PAH therapies, i.e., Bosentan (300 mg/kg/day) (Jasenovec et al., 2022). Incorporating a positive control group in future studies would strengthen the comparative assessment of EPA-L’s efficacy.

Since the lung haemodynamic is affected by PAH, systemic hypoxia develops (Porteous and Fritz, 2014). The natural physiological response to hypoxia is increased bone marrow RBC production, increased HGB level, and further increased MCHC, which is the average HGB concentration in a given RBC. These changes aim to increase the capacity of blood to absorb and carry more oxygen from the lungs. In CBC, these changes are reflected in high RBC levels, HGB, and MCHC. Concomitantly, MCV will be reduced as the newly produced RBCs are smaller. Indeed, in our research, RBC levels in the MCT-treated group were increased; however, significance was detected in comparison to basal measurement and to 0.3 mg/kg EPA-L treatment (Figure 4A). EPA-L treatment showed a trend of increase in HGB levels, and decrease in MCV levels, yet insignificantly for both parameters (Figures 4B,D). MCHC showed the most prominent change, as the MCHC levels were higher in comparison to all other groups (Figure 4C). All together, these parameters indicate a response to hypoxia, as expected due to the MCT effect (Sánchez et al., 2022; Maner BS, 2024). After EPA-L treatment, RBC, HGB, MCV, and MCHC were comparable to saline-treated rats or basal measurements. This indicates that by its effect on the pulmonary circulation, EPA-L abolished pulmonary hypoxia and, thus, its haematological consequences.

Vascular remodelling is one of the crucial events in the progression of PAH involving smooth muscle cell proliferation and an increase in intimal media thickness with narrowing of the lumen of the blood vessel, resulting in reduced blood supply to the target organs (Tobal et al., 2021). The reduction of the arterioles wall thickness and wall-lumen ratio resulting from the EPA-L treatment of the MCT-induced PAH rats indicated that EPA-L attenuated the proliferation of intimal smooth muscle cells and, thus, may affect vascular remodelling. PAH is also characterized by fibrosis and remodelling of the pulmonary arteries' extracellular matrix (ECM) with increased collagen deposition (Thenappan et al., 2018). In our study, both the low and high doses of EPA-L could not significantly preserve the tissue against MCT-induced collagen deposition in the lung tissues of the rats.

In PAH, dysfunctional bone morphogenetic protein type II receptor (BMPR-II)-mediated signalling impairs the SMAD1/5/8 pathway (Gomez-Puerto et al., 2019), whilst the overactive TGF-β pathway results in a dysregulated SMAD2/3 pathway leading to abnormal transcriptional activation of target genes (e.g., Id1 for BMP and Pai1 for TGF-β) (Tie et al., 2022). Dysregulated TGF-β signalling accelerates the remodelling of pulmonary vasculature and thus plays a pathogenic role in clinical and preclinical PAH cases (Sharmin et al., 2021; Jia et al., 2023). Several studies have shown dysregulated TGF-β/SMAD3 signalling and altered pulmonary vasculature by MCT in rats (Zakrzewicz et al., 2007; Sanada et al., 2021). Herein, both doses of EPA-L could not reverse the MCT-induced loss of SMAD3 in rats. The IHC primarily measured total SMAD3-positive cell prevalence. Thus, future analyses separating nuclear/cytoplasmic SMAD3 would be valuable to assess pathway activation.

Inflammation is one of the pathological events in PAH, and macrophage (MQ) invades the lungs in response to injury or altered homeostasis of the lungs (Zawia et al., 2021). In MCT-induced PAH models, CD68^+^ MQs accumulate in the lungs of the rats in response to tissue injury (Fan et al., 2021). The low- and high-doses of EPA-L reduced invasion of CD68^+^ MQs insignificantly in MCT-induced PAH rats. Notably, the KP1 clone has known cross-reactivity with some other myeloid cells (Gottfried et al., 2008); however, in our samples, the distribution of CD68^+^ cells (mostly alveolar and interstitial macrophages) aligns with expected macrophage localization, supporting our interpretation. Neutrophils elevation and increased lymphocytes are also indicators of inflammation (Margraf et al., 2022). In our study, MCT-induced PAH increased blood absolute neutrophil count and % lymphocyte, while EPA-L treatment decreased it, though it did not reach significance and thus failed to resolve the systemic inflammation increase in the circulation.

In PAH, the levels of tissue lipids such as ω-6 PUFAs and ω-3 PUFAs that are pro-inflammatory and anti-inflammatory, respectively, are also modulated. Research indicates that specific oxylipins, particularly eicosanoids, play significant roles in the pathophysiology of PAH. Among the oxylipins formation, AA and LA are metabolized to epoxyeicosaenoic (EET) and octadecanoic acids (EpOMEs), respectively, by cytochrome P450 (CYP) epoxygenases and further to their corresponding dihydroxy acids (HETE and DiHOMEs) by soluble epoxide hydrolase (sEH). Previous studies showed that LA (12,13-EpOME) and AA derivatives (11,12-DiHETrE) were associated with higher odds of PAH in human samples [32], and the levels of various HETE were significantly elevated compared to healthy controls [33]. Herein, the identified oxylipins were compared using the lipidomic approach before and after the treatments. The EPA-L treatment of the PAH-induced rats was found to decrease the 5,6-EET and 8,9-EET metabolites from the AA pathway and the 9- and 13-OXO-ODE from the LA pathway in comparison to the MCT-induced PH rats, while no difference was found in the HETE metabolites.

In α-LA-derived metabolites, EPA-L treatment resulted in a reduction of 9 – and 13-HOTrE. Studies have shown that 13-HOTrE reduces pro-inflammatory markers and increases anti-inflammatory cytokines (Cambiaggi et al., 2023), is associated with lower odds of PAH, and it may have protective effects or counteract inflammation in PAH (McNeill et al., 2023). In DHA-derived metabolites, 17-HDHA levels were reduced by MCT while moderately increased by EPA-L, though it did not reach significance. 17-HDHA plays a role in maintaining pulmonary arterial smooth muscle cell homeostasis, possesses anti-inflammatory effects, and suppresses the recruitment of neutrophils and monocytes/macrophages (Shu et al., 2023).

Overall, some of the anti-inflammatory eicosanoids might have decreased in MCT rats in response to the inflammatory challenge induced by MCT, while the effect of the EPA-L was inconsistent and thus did not show significant ability in resolving inflammation.

In addition to the dilation properties of EPA-L, these results suggest a role in improving PAH physiological outcomes that may overcome pulmonary artery remodelling.

## Data Availability

The raw data supporting the conclusions of this article will be made available by the authors, without undue reservation.
