# Supplementary material for: EPA-lactone derivative, 5,6-diHETE lactone, improves pulmonary arterial hypertension in a monocrotaline-induced model
Source: Front Pharmacol. 2025 Jul 10;16:1621030. doi: 10.3389/fphar.2025.1621030 (PMC12287610; doi:10.3389/fphar.2025.1621030)

**Supplementary Information**

EPA-Lactone derivative, 5,6-diHETE lactone, improves pulmonary arterial hypertension in a monocrotaline-induced model.

***Supplementary information S.1. List of oxylipins***


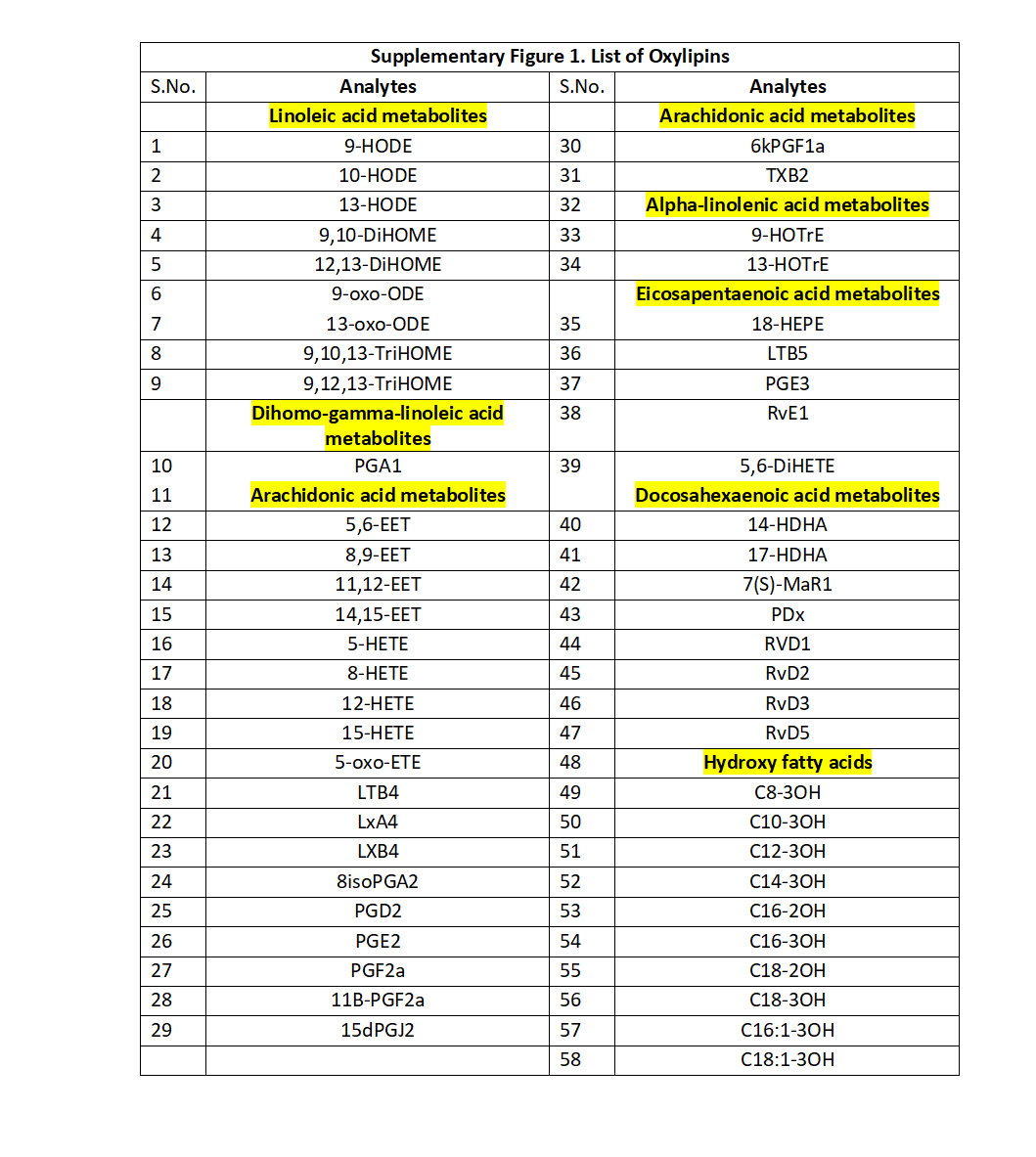

Supplement: Supplementary file 1 [file Supplementaryfile1.docx]
